# CXCR4/CX43 Regulate Diabetic Neuropathic Pain via Intercellular Interactions between Activated Neurons and Dysfunctional Astrocytes during Late Phase of Diabetes in Rats and the Effects of Antioxidant N-Acetyl-L-Cysteine

**DOI:** 10.1155/2022/8547563

**Published:** 2022-06-28

**Authors:** Dan Zhu, Tingting Fan, Yaohua Chen, Xingyue Huo, Yuping Li, Danyong Liu, Yin Cai, Chi Wai Cheung, Jing Tang, Jian Cui, Zhengyuan Xia

**Affiliations:** ^1^Department of Pain Medicine, Southwest Hospital, Army Medical University, Chongqing 400038, China; ^2^State Key Laboratory of Pharmaceutical Biotechnology, LKS Faculty of Medicine, The University of Hong Kong, Hong Kong SAR, China; ^3^Department of Anesthesiology, The University of Hong Kong, Hong Kong; ^4^Department of Population Science and Health Policy, Icahn School of Medicine at Mount Sinai, NY 10029, USA; ^5^Department of Anesthesiology, Affiliated Hospital of Guangdong Medical University, Zhanjiang, China; ^6^Department of Health Technology and Informatics, The Hong Kong Polytechnic University, Hong Kong SAR, China; ^7^Department of Anesthesiology, HuiZhou First Hospital, Guangdong Medical University, Huizhou, China

## Abstract

Growing evidence suggests that the interactions between astrocytes and neurons exert important functions in the central sensitization of the spinal cord dorsal horn in rodents with diabetes and neuropathic pain (DNP). However, it still remains unclear how signal transmission occurs in the spinal cord dorsal horn between astrocytes and neurons, especially in subjects with DNP. Chemokine CXC receptor 4 (CXCR4) plays critical roles in DNP, and connexin 43 (CX43), which is also primarily expressed by astrocytes, contributes to the development of neuropathy. We thus postulated that astrocytic and neuronal CXCR4 induces and produces inflammatory factors under persistent peripheral noxious stimulation in DNP, while intercellular CX43 can transmit inflammatory stimulation signals. The results showed that streptozotocin-induced type 1 diabetic rats developed heat hyperalgesia and mechanical allodynia. Diabetes led to persistent neuropathic pain. Diabetic rats developed peripheral sensitization at the early phase (2 weeks) and central sensitization at the late phase (5 weeks) after diabetes induction. Both CXCR4 and CX43, which are localized and coexpressed in neurons and astrocytes, were enhanced significantly in the dorsal horn of spinal cord in rats undergoing DNP during late phase of diabetes, and the CXCR4 antagonist AMD3100 reduced the expression of CX43. The nociceptive behavior was reversed, respectively, by AMD3100 at the early phase and by the antioxidant N-acetyl-L-cysteine (NAC) at the late phase. Furthermore, rats with DNP demonstrated downregulation of glial fibrillary acidic protein (GFAP) as well as upregulation of c-fos in the spinal cord dorsal horn at the late phase compared to the controls, and upregulation of GFAP and downregulation of c-fos were observed upon treatment with NAC. Given that GFAP and c-fos are, respectively, makers of astrocyte and neuronal activation, our findings suggest that CXCR4 as an inflammatory stimulation protein and CX43 as an intercellular signal transmission protein both may induce neurons excitability and astrocytes dysfunction in developing DNP.

## 1. Introduction

Diabetic neuropathy affects hundreds of million people in the world each year, and the incidence has increased dramatically over the past two to three years [1]. Diabetic neuropathic pain (DNP) refers to the clinical manifestation of diabetic peripheral neuropathy. In addition, approximately 60% of patients suffer from persistent allodynia and hyperalgesia. The clinical manifestations of DNP in patients are similar to other types of neuropathic pain, while the burning and prickling feeling is more prolonged, and the mechanism of pathogenesis is more complicated. At present, effective treatment is lacking in the clinic; and, the efficacy of tight glycemic control and the administration of ion channel-targeting drugs (e.g., gabapentin and oxcarbazepine) for the treatment of DNP are not convincing. The involvement of peripheral and central neurons could not fully explain the functional and molecular regulatory mechanisms of DNP [1].

The level of chemokine receptor CXC receptor 4 (CXCR4) is increased in the peripheral neurons of diabetic patients with neuropathy, which demonstrates a similar and crucial role for CXCR4 in the neurons of sufferers with DNP [2, 3]. CXCR4 refers to a G protein-coupled s receptor, which is primarily denoted in neurons of the spinal cord dorsal horn and dorsal root ganglion (DRG). It binds chemokine CXC motif ligand 12 (CXCL12), which can also be recognized to be stromal cell-derived factor-1 (SDF-1). [4–8]. Recently, we and others have identified that CXCR4 is strongly linked to the various responses to noxious stimuli, such as DNP and neuropathic pain triggered by sciatic nerve ligation, in the regions of DRG and spinal cord dorsal horn in rodents [9–12]. Several studies report that intraperitoneal or intrathecal administration of the AMD3100, a selective CXCR4 antagonist, can reverse nociceptive behaviors caused by partial nerve ligation-induced neuropathic pain [9, 11, 13]. However, clinically, in patients with DNP, oral administration of neuronal ion channel inhibitors could not effectively reverse mechanical allodynia and thermal hyperalgesia, suggesting the underlying involvement of glial cells in the central sensitization of the central nervous system (CNS) [14, 15].

Besides, it has also been suggested by numerous researches that the regulation of the plasticity of the central neurons (central sensitization) and primary sensory neurons (peripheral sensitization) of patients with DNP is attributable to the communication between glia and neurons [16–19]. The function and energy homeostasis of astrocytes, the most abundant type of glia in CNS, plays critical roles in maintaining the homeostasis of CNS [20]. Under basal conditions, connexin 43 (CX43), a major component of the gap junctions, is primarily expressed by astrocytes and maintains the normal shape and function of astrocytes [21, 22]. CX43 can modulate metabolism and mediate the interaction between astrocytes and neurons in the CNS [21, 23, 24]. Studies have recognized that activated astrocytes may be attributable to the central sensitization and the process of neuropathic pain in rodent models of pain, such as neuropathic pain induced by spinal nerve ligation [25–27]. However, the functional roles of CX43 hemichannels in DNP are unknown. As a result, the current work attempted to assess the expression of and the interaction between CX43 and CXCR4 in astrocytes and neurons in the spinal cord dorsal horn of DNP in rats, aiming to establish a theoretical basis for effective applications of preventive and/or therapeutic regimens for DNP.

## 2. Materials and Methods

### 2.1. Research Animals

The Laboratory Animal Unit (LAU) of University of Hong Kong provided male Sprague-Dawley (SD) rats (220-250 g), which were housed at 25°C with a 12/12 hours light-dark cycle (lights on at 7 am). In this work, the approval of animal study protocols was obtained by the Committee on the Use of Live Animals in Teaching and Research (CULATR) of the University of Hong Kong (Permit Number: 3995-16/3995-15) as well as the Laboratory Animal Welfare and Ethics Committee of Army Medical University (AMUWEC2020413). Apart from that, the protocol aimed to minimize the use of rats and avoid the animals from suffering.

### 2.2. DNP Model

A diabetic neuropathic pain model was produced by streptozotocin (STZ) (Sigma, Cat#: S0130) injection through the tail vein as previous description [28, 29]. Briefly, the anesthetization of animals was performed with the mixture of ketamine (67.7 mg/kg) and xylazine (6.77 mg/kg) delivered via intraperitoneal injection. Diabetes was induced in rats via the single intravenous injection of 65 mg/kg STZ, which was dissolved with citrate buffer (pH 4.5). Besides, after the completion of STZ injection, the levels of blood glucose were assessed with a glucometer (OneTouch Ultra) 72 hours. Besides, rats which had a blood glucose level higher than 16.7 mmol/L were considered to be diabetic, which can also be included in the ensuring study. At one week after inducing diabetes and onwards, the paw withdrawal threshold (PWT) as well as the paw withdrawal latency (PWL) were evaluated with the purpose of evaluating heat hyperalgesia as well as mechanical allodynia.

### 2.3. Experimental Protocol

**Figure 1:**
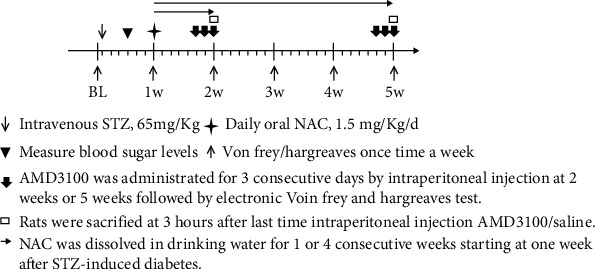
Timeline of experiment protocol in SD rats. BL: baseline; w: week.

This study conducted two types of research to firstly investigate the localization and expression levels of CXCR4 in the spinal cord dorsal horn and then to detect the impacts of the CXCR4 antagonist AMD3100 (Sigma, A5602) and the treatment impacts of N-acetyl-L-cysteine (NAC) (Sigma, A7250) on DNP. In the first part of study, animals were classified to two groups (*n* = 6/group) at random: (1) the control group (saline group, i.v. injection of saline) and (2) diabetes group (Dia): rats were injected with STZ. For the second part study, the rats were randomly allowed to one of the four groups (*n* = 6, per group): (1) the saline control group: the injection of nondiabetic rats were performed with saline (i.p.); (2) Dia group: untreated diabetic group; (3) AMD3100-treated diabetic group (AMD group): rats with diabetes were administered with AMD3100 (5 mg/kg/d, i.p.) for three consecutive days at 2 weeks or at 5 weeks after the establishment of type 1 diabetes following the injection of STZ. Diabetic rats were euthanized either at 2 weeks or at 5 weeks after the completion of AMD3100 treatment and related tests. (4) NAC-treated diabetic rats (NAC group): NAC was given daily via drinking water to rats with diabetes at a dosage of 1.5 mg/kg/d. As previously described, the chemical NAC was diluted in the drinking water and was given for 4 consecutive weeks beginning from week 1 after intravenous STZ injection [30]. Three consecutive days of intraperitoneal injection of AMD3100 in rats has been shown to effectively inhibit CXCR4 [31]. Behavioral tests at baseline and the behavioral changes over time during the course of treatment were assessed before and after intravenous STZ injection. The timeline of the experimental protocol in rats is shown in Figure 1.

### 2.4. Behavioral Test

Before intravenous injection of STZ, heat hyperalgesia and mechanical allodynia were evaluated by behavioral tests as the baseline. At the same time, the rats' behavioral changes were examined at week 2 and week 5 after the establishment of diabetes following STZ administration. As we and others reported previously, bilateral hind paw withdrawals were considered a positive reaction for the establishment of DNP in an animal model [10, 32].

### 2.5. Mechanical Allodynia

As described previously [10, 33, 34], the PWT of mechanical allodynia was examined by adopting a specific apparatus for Von Frey test (IITC/Life Science, Inc., USA). In short, rats with individually assigned numbers were positioned separately in the plexiglass boxes with a metal mesh floor and were also permitted a quarter hour for the animals with the purpose of adapting to the experimental environment. With a blunted probe, hind paws were vertically and carefully stimulated to elicit positive paw withdrawal. The rest was repeated for 3 times in each paw, and the value was averaged. In addition, the mean was recorded to be the mechanical threshold.

### 2.6. Thermal Hyperalgesia Test

Using a Hargreaves apparatus (Ugo Basile, Varese, Italy), thermal hyperalgesia was measured. Before the test, rats in each group were individually placed in cages for 15 minutes to adapt to the environment. A free heat source was placed below the plexiglass plate to give a noxious heat stimulus, and the PWL was recorded. The maximum PWL of 20 s and 5-minute intervals in each trial were set to avoid excessive noxious heat source stimulation, and the mean value of 3 consecutive trials was calculated for each hind paw.

### 2.7. Tissue Preparation

The euthanization of rats was performed with overdose sodium pentobarbital (100 mg per kilogram of body weight). The L3-L5 spinal cord at both sides were obtained at two time points, respectively, at early phase (2 weeks) and at late phase (5 weeks). After being quickly removed, the tissue samples were frozen in liquid nitrogen and also housed at -80°C in order to carry out western blot assays. In terms of immunofluorescence assay, rats were initially perfused with normal saline with the application of 4% paraformaldehyde (PFA) (Sigma, Cat#: 8187151000). Subsequently, the L3-L5 segments of the spinal cord were then postfixed in 4% PFA and stayed for the whole night at 4°C as well as dehydrated in 30% sucrose solution.

### 2.8. Primary Cell Cultures

Using a protocol previously detailed, spinal cord neuron cultures were prepared from postnatal day 1 SD rats (Centre for Comparative Medicine Research, the University of Hong Kong) [35]. Briefly, isolated spinal cords from 4 to 6 SD rats were rapidly minced and rinsed three times with the Dulbecco's Modified Eagle Medium and Nutrient Mixture F-12 (DMEM/F12, GIBCO, Cat#: A4192001), and digested for a duration of 25 minutes at 37°C in a solution containing 2 mg/ml papain (Sigma, Cat#: P3152) and 1 *μ*g/ml DNAse (Sigma, Cat#: 260913) in neurobasal medium (GIBCO, 21103049). The termination of enzyme reaction was made by the supplementation of 10% fetal bovine serum (FBS) (GIBCO, Cat#:16140063) with DMEM/F12. Tissue pieces were triturated gently in 1 ml cold culture medium (DMEM/F12 added with 10% FBS). Then, the resulting suspension was filtered using the 70 *μ*m cell strainer (BD Falcon, Cat#: 352350) and be centrifuged at 1,000 rpm for a duration of 5 minutes. In addition, the cells were seeded into a culture flask as well as placed in 37°C aseptic incubators with 5% CO_2_. After 40 minutes, flipped the culture flask, then aspirated the cell suspension, resuspended in DMEM/F12 medium, and plated at 10,000 cells/ml density in a confocal 35-mm petri dish (Solarbio, YA0572). After 4 to 6 hours, when the cells were almost attached to the petri dish, the medium was changed to neurobasal (Invitrogen, Cat#: 21103049) culture medium supplemented with B27 (Invitrogen, Cat#: 17504044). Twice each week, half of the culture medium was substituted with fresh medium. Using an OLYMPUS microscope BX46, the viability of neurons was confirmed after being placed for 5 days in culture.

Spinal cord astrocyte culture was conducted following a protocol described previously [36]. Briefly, isolated and minced spinal cord tissues from 4 to 6 SD rats were digested with 0.125% trypsin (Sigma, Cat#: T4049) solution and were stayed for a duration of 15 minutes at 37°C, and the tissues were then gently triturated in DMEM/F12 containing 10% FBS. With a 70 *μ*m cell strainer, the resulting suspension was filtered. Then, the cells were resuspended in DMEM/F12 culture medium which was housed at 37°C in aseptic incubators with 5% CO_2_. After 24 hours, the cells, at a density of 10,000 cells, were seeded in 35-mm confocal petri dishes in DMEM/F12 culture medium without FBS. Twice each week, half of the culture medium was substituted with fresh medium. Astrocyte viability was confirmed using an OLYMPUS microscope BX46 after being placed in culture for 5 days.

### 2.9. Western Blot Analysis

The harvested spinal cord tissue was homogenized using a Polytron homogenizer (Kinematica, Switzerland) using ice-cold lysis buffer in the volume of in 100 *μ*l and for a duration of 1 hour. The centrifugation of lysate was further performed at 12,000 rpm for a quarter hour at 4°C. After extraction and centrifugation, the assessment of protein concentrations was made by adopting the Bradford Protein Assay Kit (Bio-Rad, USA). In addition, proteins at the amounts of 30 *μ*g per sample were divided using 12.5% SDS-polyacrylamide gels and were then transferred to polyvinylidene fluoride (PVDF) membranes. After being blocked with 5% nonfat milk that was dissolved in Tris-buffered saline with Tween (TBST) for 1 hour under the condition of chamber temperature, the membranes were then raised with rabbit anti-CXCR4 (1 : 1000, Abcam, ab124824), mouse anti-CX43 (1 : 1000, Sigma, C8093), mouse anti-GFAP (1 : 1000, Cell Signaling, Cat#: 3670), and mouse anti-c-fos (1 : 1000, Abcam, ab208942) antibodies overnight under the condition of 4°C. Thereafter, the membranes were rinsed for 3 times using TBST (10 min each), before being nurtured with horseradish peroxidase (HRP)-linked antirabbit IgG and antimouse IgG secondary antibodies (1 : 2000, Cell Signaling, A0545/SAB5600195) for an hour under the condition of room temperature. In addition, the bands of proteins were visualized by incubating the membranes in ECL solution (Bio-Rad, Cat#: 1705061) for 1 minute and subject to X-ray films (Kodak, USA) in the dark room. Apart from that, we adopted the ImageJ software (the National Institutes of Health, USA) for assessing optical density.

### 2.10. Immunofluorescence Staining

As described previously, immunofluorescence staining of spinal cord slices was performed [10]. In brief, 20 *μ*m-thick slices of spinal cord L3-L5 were cut by using a Cryotome (Thermo, USA). The permeabilization of sections was made by incubation with 0.25% Triton X-100 for a duration of 15 minutes and subsequently was blocked with the concentration of 10% bovine serum albumin (BSA) (Sigma, Cat#: 10735108001) for a duration of 1 hour under the condition of the room temperature. Subsequently, the slices of the spinal cord dorsal horn at L3-L5 were incubated with combinations of the rabbit anti-CXCR4 (1 : 200, Abcam, ab124824) and the mouse anti-c-fos (1 : 200, Abcam, ab208942), rabbit anti-CXCR4 and mouse anti-GFAP (1 : 200, Cell Signaling, Cat#: 3670), rabbit anti-CXCR4 and mouse anti-CX43 (1 : 200, Sigma, C8093), or mouse anti-CX43 (1 : 200, Sigma, C8093) antibodies overnight at 4°C. In addition, after three washes (10 minutes each time), slices were illuminated with a mixture of Alexa Fluor 488-conjugated antimouse (1 : 500, Abcam, ab150113) and Alexa Fluor 568-conjugated antirabbit (1 : 500, Abcam, ab175703) antibodies or Alexa Fluor 568-conjugated antirabbit antibody alone for the duration of 1 hour at room temperature. Subsequently, the nuclei subsequently stained using DAPI (Cell Signaling, Cat#: 8961). In addition, images were captured with the use of an LSM 710 laser scanning confocal microscope (Zeiss) and were then explored by adopting the ImageJ software.

Immunofluorescence staining of primary cultured astrocytes and neurons in confocal 35-mm petri dishes was carried out as the description presented in vivo. Briefly, cultured cells were fixed by using 4% PFA for the duration of 30 minutes at 37°C and permeabilized using 0.1% Triton X-100 for a quarter hour. Next, we blocked the cells with 1-hour exposure to 10% BSA in PBS. Subsequently, the astrocytes were incubated with a combination of rabbit anti-CXCR4 (1 : 200, Abcam, ab124824) and mouse anti-CX43 (1 : 200, Sigma, C8093) or rabbit anti-CXCR4 (1 : 200, Abcam, ab124824) and mouse anti-GFAP (1 : 200, Cell Signaling, Cat#: 3670) antibodies. Primary neurons were incubated with a combination of rabbit anti-CXCR4 (1 : 200, Abcam, ab124824) and mouse anti-CX43 (1 : 200, Sigma, C8093) or rabbit anti-CXCR4 (1 : 200, Abcam, ab124824) and mouse anti-NeuN (1 : 200, Abcam, ab104224) antibodies through the whole night at 4°C. On the next day, secondary antibodies, a mixture of Fluor 488-conjugated antimouse (1 : 500, Abcam, ab150113) and Alexa Fluor 568-conjugated antirabbit (1 : 500, Abcam, ab175703) antibodies were added to the petri dishes and were then incubated for a duration of an hour at chamber temperature, and the nuclei were stained with DAPI, with images being analyzed using the confocal laser microscope (LSM 800, Zeiss).

### 2.11. Assays for the Antioxidant Enzymes Glutathione Peroxidase (GSH-Px) and Superoxide Dismutase (SOD) and the Lipid Peroxidation Product Malondialdehyde (MDA)

In this study, the GSH-Px (S0057S, Beyotime Institute of Biotechnology, Nantong, Jiangsu, China), MDA (S0131S, Beyotime Institute of Biotechnology, Nantong, Jiangsu, China), and SOD (S0109, Beyotime Institute of Biotechnology, Nantong, Jiangsu, China) Detection Kits were, respectively, employed to measure GSH-Px (nm/mgprot), MDA (nmol/mgprot), and SOD (U/mgprot) levels in the spinal cord tissues. The assay procedures followed the respective supplier's instructions to calculate the content or activity of the samples.

### 2.12. Statistical Analysis

Data are denoted as mean ± the standard deviation (SD). Besides, the same time point values of PWT and PWL among groups were analyzed by the application of one-way ANOVA with Tukey's post hoc test, while comparisons of the changes of PWT and PWL over time were assessed by paired *t*-test. For molecular data, the results were explored by adopting one-way ANOVA based on Tukey's post hoc test. In addition, the SPSS 19.0 (USA) and the GraphPad Prism 7.0 (USA) statistical software were applied to perform data analyses. A *P* value less than 0.05 was considered to show statistically significant difference.

## 3. Results

### 3.1. Changes of c-fos, CXCR4, and GFAP Protein Expression at the L3-L5 Level Spinal Cord of Diabetic Rats at 2 Weeks and 5 Weeks of the Disease

**Figure 2:**
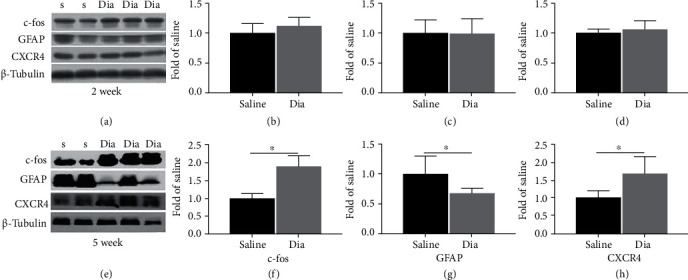
Streptozotocin (STZ)-induced DNP in rats enhanced spinal cord dorsal horn CXCR4 and c-fos and decreased GFAP at 5 weeks (e–h) yet not at 2 weeks (a–d) of diabetes. (a, e) c-fos, GFAP, and CXCR4 expression in the spinal cord, according to western blotting, at 2 and 5 weeks after intravenous STZ-induced diabetes (Dia) and at 2 and 5 weeks after injection of saline (saline control group). (b–d, f–h) Quantification of c-fos, GFAP, and CXCR4 levels in the spinal cord. Apart from that, western blot results can be shown to be means ± SD. ∗*P* < 0.05 in relative to saline group, *n* = 6/group. S = saline; Dia = diabetes.

As shown in Figure 2, the protein levels of the proinflammatory CXCR4 in the spinal cord was significantly increased at week 5 (late phase), but not at week 2 (early phase) of diabetes, which is in line with our previous report [10]. However, the expression of the astrocytic marker GFAP and the neuronal activation marker c-fos in the spinal cord dorsal horn of rats with DNP in the early and late phases of diabetes has not been explored previously. As shown in Figure 2, our current study demonstrated that the protein levels of spinal cord c-fos and CXCR4 were significantly enhanced at 5 weeks (Figures 2(f) and 2(h), *P* < 0.05) but not at 2 weeks (Figures 2(b) and 2(d)). In particular, in the late phase (5 weeks), GFAP protein contents (Figure 2(g)) decreased significantly in rats with DNP, which is in contrast to the observations in rats in other models of neuropathic pain (e.g., neuropathic pain trigged by chronic constriction injury) [37].

### 3.2. Localization and Expression of CXCR4 in the Spinal Cord Dorsal Horn and in Primary Cultured Astrocytes and Neurons

**Figure 3:**
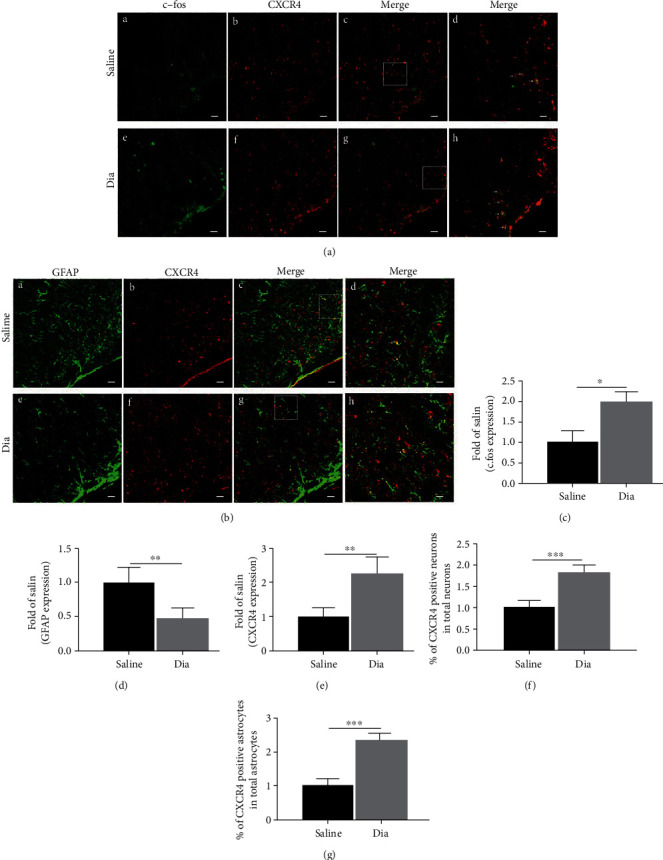
Confocal images showed coexpression and distribution of CXCR4 and GFAP, CXCR4 and c-fos, and the expression of c-fos, CXCR4, and GFAP in the L3-L5 spinal cord dorsal horn in rats with STZ-induced DNP at 5 weeks of diabetes and in control rats receiving saline at 5 weeks. (a, b) Double-immunostaining for c-fos (green (a) (A and E)), GFAP (green (b) (A and E)), and CXCR4 (red, B and F) in the spinal dorsal horn after STZ-induced diabetes at 5 weeks. C and G were merged images of A and B or E and F separately (original magnification: 200×, scale bar A–C, E–G 20 *μ*m). Merged and enlarged images were shown in D and H (original magnification: 400×, scale bar 10 *μ*m). Immunostaining for c-fos (a) (A, E), GFAP (b) (A, E), and CXCR4 (B, F) of the spinal cord dorsal horn was determined at 5 weeks after STZ-induced diabetes. Besides, quantitative analysis of c-fos (c), GFAP (d), and CXCR4 (e) of the intensities at 5 weeks. Quantification of the coexpression of CXCR4 and c-fos (f) and CXCR4 and GFAP (g). All data are indicated to be means ± SD. ∗*P* < 0.05, ∗∗*P* < 0.01, and ∗∗∗*P* < 0.001 in relative to saline group. *n* = 6/group. .

**Figure 4:**
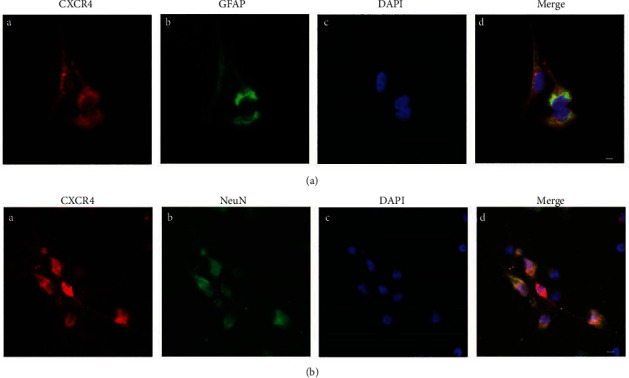
Representative confocal images presented co-expression of CXCR4 and GFAP, CXCR4 and NeuN in primarily cultured astrocytes and neurons, respectively. (a, b) Double-immunostaining for CXCR4 (red, A), GFAP (astrocytic marker, green (a) (B)), NeuN (neuronal marker, green (b) (B)), DAPI (nuclear marker, blue, C). (a) (D) and (b) (D) were merged A, B, and C images (original magnification: 400×, scale bar 10 *μ*m, *n* = 6/group).

In terms of control and diabetic rats, immunofluorescence staining of the L3-L5 spinal cord sections confirmed that the expression of CXCR4 mainly existed in neurons and resided partly in astrocytes (Figures 3(a) and 3(b)). The expression of CXCR4 was notably enhanced in diabetic rats in relative to that in the control rats (*P* < 0.01, saline vs. Dia, Figure 3(e)). Furthermore, the immunostaining intensity of c-fos was significantly enhanced in rats with DNP (*P* < 0.05, saline vs. Dia, Figure 3(c)). The coexpression of CXCR4 and c-fos marked active neurons was also obviously enhanced in diabetic rats in comparison with that in the control rats (*P* < 0.001, saline vs. Dia, Figure 3(f)). Notably, immunostaining intensity of GFAP was notably reduced in DNP rats (*P* < 0.01, saline vs. Dia, Figure 3(d)), whereas the coexpression of CXCR4 and GFAP-marked astrocytes were notably enhanced in the spinal dorsal horn of rats with DNP (*P* < 0.001, saline vs. Dia, Figure 3(g)). The results of primary cultured neurons and astrocytes consolidated that CXCR4 was colocalized with GFAP-marked astrocytes and NeuN-marked neurons (Figures 4(a) and 4(b)). Collectively, the obtained data indicate that CXCR4 can be denoted in both neurons and astrocytes in the spinal cord dorsal horn and that the expression of CXCR4 is enhanced in neurons and astrocytes in rats with DNP.

### 3.3. Heat Hyperalgesia and Mechanical Allodynia Development in Diabetic Rats and the Treatment Effects with AMD3100 or NAC at 2 and 5 Weeks of Diabetes

**Figure 5:**
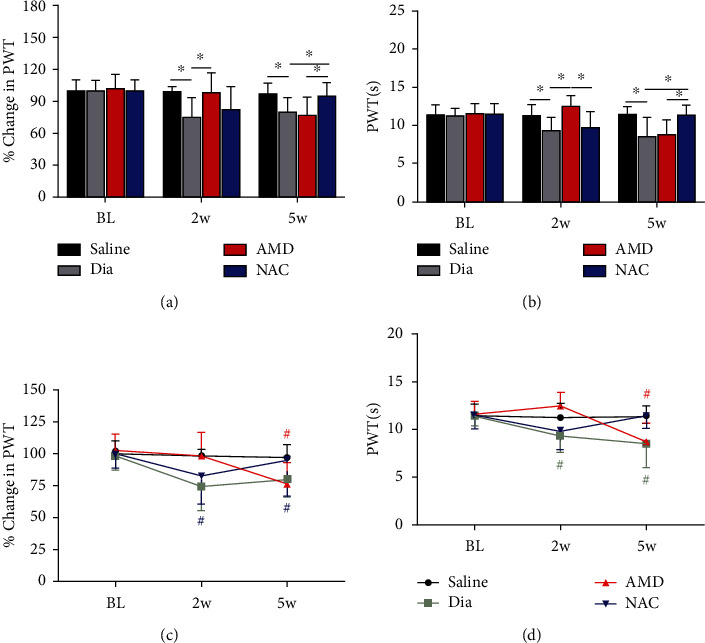
Variations of PWT (a) and PWL (b) in untreated diabetic rats (Dia) and in nondiabetic control (saline) or diabetic rats exposed to treatment with AMD or NAC and variations of PWT (c) and PWL (d) from baseline in the saline, Dia, AMD, or NAC group. PWT (a) and PWL (b) were notably decreased in STZ-induced diabetic rats and increased in AMD-treated diabetic rats but not in NAC-treated diabetic rats at 2 weeks, while PWT and PWL were significantly increased in diabetic rats receiving oral NAC by 5 weeks as identified by electronic Von Frey and Hargreaves test. In addition, PWT (c) and PWL (d) were reduced from 2 weeks to 5 weeks compared to baseline in Dia group, and PWT (c) and PWL (d) were obviously lowered in 5 weeks in comparison with baseline in the AMD group. All findings are shown to be means ± SD, *n* = 6/group, ∗*P* < 0.05. ^#^*P* < 0.05 vs. baseline. BL = baseline; w = week; PWT = paw withdraw threshold; PWL = paw withdrawal latencies; Dia = diabetes; NAC = N-acetyl-L-cysteine; AMD = AMD3100.

To explore the heat hyperalgesia and mechanical allodynia development in rats undergoing DNP and the impacts of treatment with AMD3100 or NAC, mechanical allodynia and heat hyperalgesia were evaluated as did in our previous DNP model [10]. The baseline values of PWL and PWT did not show the difference among groups (Figures 5(a) and 5(b), (BL) *P* > 0.05). Besides, 14 days after the establishment of diabetes, the PWT and PWL values were notably lowered in the diabetic rats in comparison with rats in the nondiabetic control group (Figures 5(a) and 5(b), *P* < 0.05, Dia vs. saline), while AMD3100 treatment reversed the reductions in PWT and PWL seen in the untreated diabetic group (Figures 5(a) and 5(b), *P* < 0.05, Dia vs. AMD). However, NAC did not obviously affect the PWT and PWL values in rats after 2 weeks of diabetes (Figures 5(a) and 5(b), *P* > 0.05, Dia vs. NAC). At 5 weeks of diabetes, 4 weeks of continuous NAC treatment that was initiated at 1 week of diabetes induction significantly increased both PWT and PWL in diabetic rats to levels comparable to those of the nondiabetic rats (Figures 5(a) and 5(b), *P* < 0.05, Dia vs. NAC). However, unexpectedly, AMD3100 intraperitoneal injection at 5 weeks of diabetes did not have a significant effect on PWT or PWL (Figures 5(a) and 5(b), *P* > 0.05, Dia vs. AMD). Furthermore, the PWT and PWL values were obviously reduced from 2 weeks to 5 weeks as compared to baseline in diabetic group. And PWT and PWL were not significantly changed at 2 weeks compared to baseline in the AMD group but significantly decreased at 5 weeks, suggesting a positive treatment result in early phase (Figures 5(c) and 5(d), *P* < 0.05, BL vs. 2 weeks, 5 weeks).

### 3.4. Changes of the c-fos, CXCR4, and GFAP Proteins in the L3-L5 Spinal Cord and the Treatment Effects of AMD3100 or NAC at 2 and 5 Weeks of Diabetes

**Figure 6:**
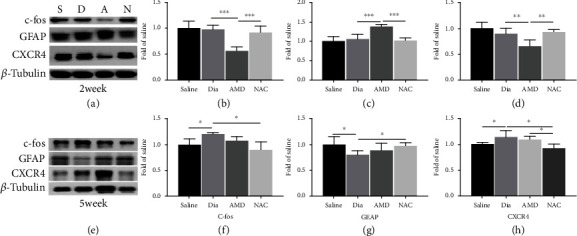
Correlative proinflammatory protein CXCR4 and neuronal marker protein c-fos of the spinal cord in rats with DNP was inhibited, and astrocytic marker protein GFAP was activated by intraperitoneal AMD3100 at 2 weeks and by daily oral NAC treatment at 5 weeks of diabetes. (a, e) Western blot demonstrated the protein expression of c-fos, GFAP, and CXCR4. (b–d, f–h) Quantitative analysis of c-fos, GFAP, and CXCR4 comparing with *β*-tubulin in spinal dorsal at 2 weeks and 5 weeks. In addition, all the obtained data can be shown to be means ± SD. ∗*P* < 0.05, *n* = 6/group.

Based on the behavioral results, we further examined the expressions of c-fos, CXCR4, and GFAP proteins in the spinal cord of the animals in the four experimental groups. Notably, in the 2-week diabetic group (Dia), the spinal cord expression levels of c-fos, CXCR4, and GFAP did not present obvious difference from that in the nondiabetic control group (Figure 6(a)), which were not consistent with the behavioral results of PWT and PWL (Figures 5(a) and 5(b)). As a previous study demonstrated, in STZ-induced diabetic rats, the proinflammation proteins TNF-*α* and CXCR4 were both enhanced at the initial phase (2 weeks) of diabetes in the DRG but did not change in the spinal cord dorsal horn [10]. Therefore, peripheral sensitization of DNP rats occurred at an early stage, and prolonged peripheral sensitization might trigger central sensitization of the spinal cord dorsal horn [10]. AMD treatment significantly hindered the expression of CXCR4 and c-fos in the spinal cord (Figures 6(b) and 6(d), *P* < 0.05, Dia vs. AMD) at 2 weeks. GFAP protein expression increased at an earlier phase following AMD3100 treatment (Figure 6(c), *P* < 0.05, Dia vs. AMD), but no remarkable changes were seen at the later phase (Figures 6(f)–6h, *P* > 0.05, Dia vs. AMD). Furthermore, at 5 weeks, the expression of c-fos and CXCR4 in the spinal cord could be reduced by daily oral administration of NAC (Figure 6(f) and 69h), *P* < 0.05, Dia vs. NAC), whereas GFAP protein (Figure 6(g), *P* < 0.05, Dia vs. NAC) was increased. Nevertheless, CXCR4, c-fos and GFAP protein did not alter significantly in diabetic rats at 2 weeks nor did NAC have a significant impact on CXCR4, c-fos, and GFAP protein levels at this stage (Figures 6(b)–6(d), *P* > 0.05, Dia vs. NAC). The above data demonstrated that dysfunctional astrocytes might promote and maintain central sensitization and exert an essential function in the late phase of STZ-induced diabetic rats. Moreover, daily oral administration of NAC improved nociceptive behaviors in the late stage (5 weeks of diabetes) as assessed by PWT and PWL and significantly reduced the expression of CXCR4 in the spinal cord dorsal horn of rats with DNP (Figure 5, *P* < 0.05, Dia vs. NAC; Figure 6(h), *P* < 0.05, Dia vs. NAC).

### 3.5. NAC Inhibited the Accumulation of Oxidation Products

**Figure 7:**
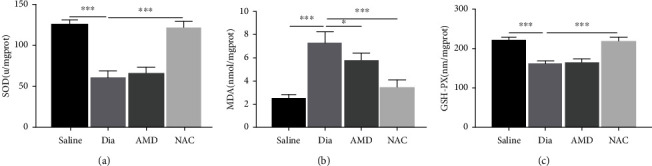
Changes of antioxidant enzymes (SOD and GSH-PX) and lipid peroxidation product malondialdehyde (MDA) in the spinal cord in rats undergoing DNP at 5 weeks of diabetes and the treatment effects of NAC and AMD. (a–c) The activity of SOD (a) and the contents of MDA (b) and GSH-Px (c) in rat spinal cord at 5 weeks of diabetes. Besides, all the obtained data are denoted to be means ± SD. ∗*P* < 0.05, ∗∗*P* < 0.01, and ∗∗∗*P* < 0.001, *n* = 6/group.

We further examined the antioxidant effect of NAC. As shown in Figure 7, a significant rise was found in MDA contents of spinal cord at 5 weeks after STZ-induced diabetes (Figure 7(b), *P* < 0.001, Dia vs. saline) accompanied by reduced activities of SOD and GSH-Px (Figures 7(a) and 7(c), *P* < 0.001, Dia vs. saline). Furthermore, NAC treatment significantly prevented the above changes in MDA, SOD, and GSH-Px expression (Figures 7(a)–7(b), *P* < 0.001, Dia vs. NAC). As shown in Figure 7(b), AMD3100 moderately yet obviously reduced the accumulation of MDA (*P* < 0.05, Dia vs. AMD); however, it did not increase the antioxidant enzymes SOD and GSH-Px (*P* > 0.05, Dia vs. AMD). These results illustrated that NAC enhanced the ability to combat against oxidative damage and increased endogenous antioxidant capacity, and AMD3100 only demonstrated the ability to inhibit oxidative damage in the spinal cord at late phase (week 5) after STZ-induced diabetes.

### 3.6. Expression and Localization of CX43 in the Spinal Cord, and Coexpression of CXCR4 and CX43 in Spinal Cord Dorsal Horn and in Primary Culture Astrocytes/Neurons

**Figure 8:**
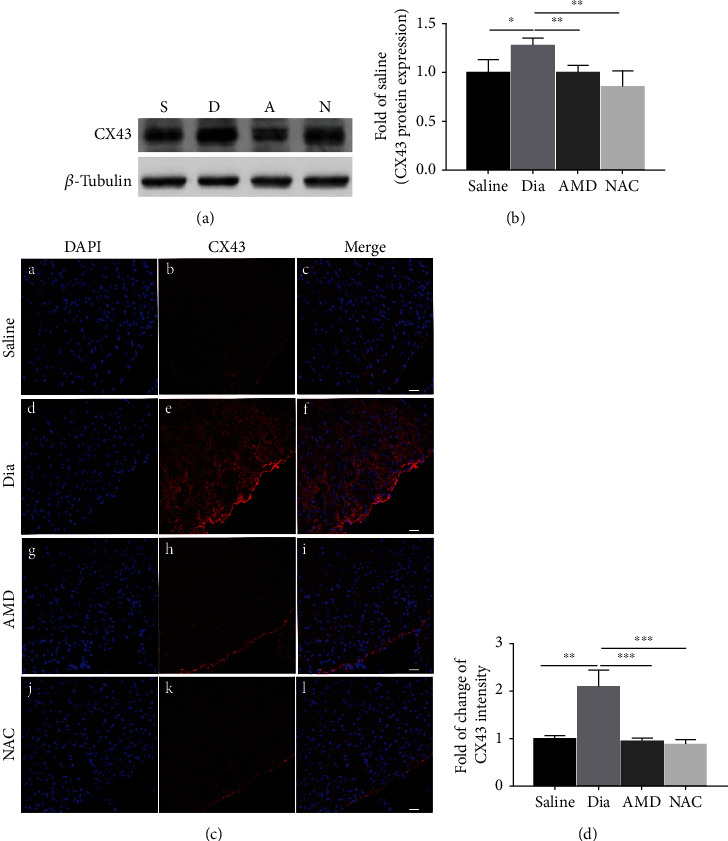
DNP was associated with significantly added expression of CX43 in the spinal cord dorsal horn of STZ-induced diabetic rats at 5 weeks of the disease, which could be inhibited by intraperitoneal AMD3100 and NAC. (a) Western blot and (c) confocal images showed the protein expression of CX43 in the spinal cord dorsal horn. (b) Quantitative analysis of CX43 comparing with *β*-tubulin in the spinal dorsal at 5 weeks. All the data are demonstrated to be means ± SD. ∗*P* < 0.05, ∗∗*P* < 0.01, and ∗∗∗*P* < 0.001, *n* = 6/group. C, F, I, and M were merged images A and B, D and E, G and H, and J and K, separately. (d) Semiquantification of CX43 immunofluorescence intensity. *n* = 6/group (original magnification: 200×, scale bar 20 *μ*m).

**Figure 9:**
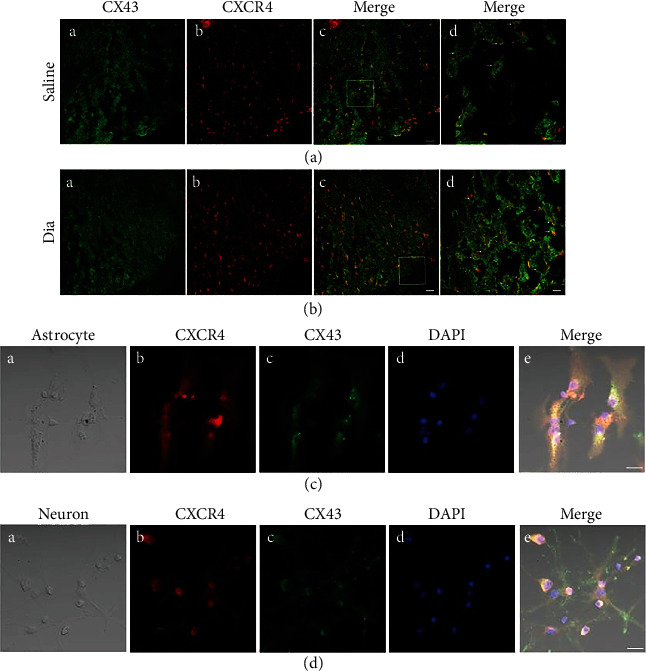
Confocal images show coexpression of CXCR4 and CX43 in the spinal dorsal horn of rats after STZ-induced DNP at 5 weeks of diabetes and in saline control rats (a, b), and in primary culture astrocytes (c) and neurons (d). (a) and (b) co-expression of CX43 and CXCR4 of spinal dorsal cord were shown in confocal images. (a) (A, B) and (b) (A, B) were double-immunostaining for CX43 (green, A) and CXCR4 (red, B). (a) (C) and (b) (C) were merged images A and B (original magnification: 200×, scale bar 20 *μ*m). Merged and enlarged images were shown in (a) (D) and (b) (D) (original magnification: 400×, scale bar 10 *μ*m, n = 6/group). (c) (A) and (d) (A) were bright field images of astrocytes and neurons, respectively. (c, d) Double-immunostaining for CXCR4 (red, B), CX43 (green, C), DAPI (blue, D). (c) (E) and (d) (E) were merged images A, B, C, and D (original magnification: 400×, scale bar 20 *μ*m, *n* = 6/group).

Numerous researches have demonstrated that astrocytes exert essential functions in energy metabolism, not only in providing nutritional support for neurons in the CNS but also in maintaining the dynamic balance of the microenvironment [16, 38, 39]. CX43, a hemichannel protein that primarily exists in astrocyte membranes and is permeable to Ca^2+^, IP_3_, ATP, cAMP, etc. [40–42], acts as a “bridge” channel for intercellular connections. To elucidate the cause and effect of astrocyte dysfunction on the central sensitization of rats with DNP in the late phase of diabetes, this study further explored the levels of CX43 hemichannel protein in the L3-L5 spinal cord dorsal horn. Contrary to our expectation, as shown in Figures 8(a) and 8(c), CX43 expression in diabetic rats increased significantly at the late phase, and either AMD3100 intraperitoneal injection or daily oral administration of NAC reversed this increase (Figures 8(b) and 8(d)). Furthermore, immunostaining confirmed that the proinflammatory protein CXCR4 and hemichannel protein CX43 were coexpressed in the spinal cord dorsal horn, both in rats with DNP and in saline-treated nondiabetic control rats (Figures 9(a) and 9(b)). With the purpose of further exploring the coexpression of these proteins in the spinal cord dorsal horn, we used immunostaining to locate the CXCR4 and CX43 proteins in primary cultured neurons and astrocytes. Immunostaining showed that CXCR4 and CX43 were coexpressed in both primary neurons and astrocytes (Figures 9(c) and 9(d)). All these results suggested that activated CX43 and CXCR4 could affect the development of DNP in STZ-induced type 1 diabetic rats via dysfunctional astrocytes and activated neurons in the spinal cord dorsal horn at the late phase of diabetes.

## 4. Discussion

The current study revealed that neuronal activation and astrocyte dysfunction may be mediated by upregulated CXCR4 and CX43 in the spinal cord dorsal horn of STZ-induced diabetic rats at the late phase of the disease which may represent a mechanism of DNP development. We dissected the potential mechanism of DNP using several approaches: (1) in diabetic rats, the STZ-induced DNP model could persistently elicit the upregulation of CXCR4 in both neurons and astrocytes in the spinal cord dorsal horn (from 2 weeks to 5 weeks). (2) Activated neurons and dysfunctional astrocytes could be observed in the spinal cord dorsal horn of rats with DNP at late phase diabetes (5 weeks) yet not in the early phase (2 weeks). (3) Intraperitoneal administration of AMD3100 could reverse the thermal hyperalgesia and mechanical allodynia of STZ-induced DNP in rats and inhibited CXCR4 protein expression in the spinal cord dorsal horn in the early phase, but not in the late phase of DNP. Comparatively, NAC given via daily oral administration to rats with DNP maintained the behavioral responses as evaluated by PWT and PWL at a level that was comparable to that seen in the nondiabetic control rats and downregulated CXCR4 in the late phase of diabetes. (4) The expression of the hemichannel protein CX43, which mediates neuron-glia interactions, was notably upregulated in the spinal cord dorsal horn of rats with DNP in the late phase of diabetes, and intraperitoneal administration of AMD3100 inhibited the upregulation of CX43 and attenuated symptoms of DNP, which further illustrates the interactions between dysfunctional astrocytes and activated neurons in the development of DNP. (5) Coexpression of CX43 and CXCR4 was confirmed in the spinal cord dorsal horn of DNP rats, as well as in the primary cultures of astrocytes and neurons isolated from neonatal nondiabetic rats.

### 4.1. CXCR4 in the Spinal Cord Dorsal Horn Astrocytes and Neurons in the Early- and Late-Phase Diabetic Rats with Neuropathic Pain

Intravenous administration of STZ has been considered a more reliable and reproducible method to induce diabetic rats with neuropathic pain than intraperitoneal administration of STZ [43]. The dose of intravenous STZ at 65 mg/kg induces type 1 diabetes in rats reproducibly [9, 10, 29]. Notably, as previous studies pointed out, heat hyperalgesia and mechanical allodynia occurred in the first week in STZ-induced diabetic rats [10, 44]. CXCR4 as well as its ligand SDF-1 were found to be upregulated in the spinal cord dorsal horn of animals with neuropathic pain triggered by partial sciatic nerve ligation (PSNL)- and in chronic postischemia (CPIP)-induced neuropathic pain [9, 11]; however, both of them are inhibited by AMD3100. Therefore, we specifically assessed the spinal cord dorsal horn CXCR4 variations in rats at the early and late phases of diabetes. Our previous study revealed that the CXCR4 was enhanced only in the DRG neurons at week 2 (early phase of diabetes) after STZ injection [10]. Furthermore, CXCR4 protein was enhanced in both the DRG and the spinal cord dorsal horn at week 5 (late phase of diabetes), which was consistent with the upregulation of proinflammatory protein expression in other neuropathic pain models (e.g., cancer pain model, nerve ligation) [45, 46]. This result suggested that persistent peripheral inflammatory stimulation induced central sensitization in DNP.

Accumulating studies indicated that astrocytes exert essential functions in the maintenance of neuropathic pain. Especially, reactive astrocytes have been revealed in some animal models of neuropathic pain such as pain triggered by chronic constriction injury (CCI), suggesting that reactive astrocytes could modulate neuropathic pain. Our finding revealed an obvious decrease of GFAP expression in the spinal cord dorsal horn of diabetic rats, showing consistence with previous studies [47–49]. This pathological change of astrocytes in DNP was different from reactive astrocytes that was characterized by increased GFAP expression [9, 36]. GFAP, as the main intermediate filament protein in mature astrocytes, also serves as a critical constituent of the cytoskeleton in astrocytes during the development, and its decreased expression could result in the failure of astroglial support, impaired homeostatic function of astrocytes, and the abnormal synaptic transmission [50]. This pathological change of astrocytes has been identified in various neurological disorders such as neuropsychiatric disorders, addictive disorders, epilepsy, and neurodegenerative degeneration [50], which may also be a critical pathogenesis of DNP. In addition, astrocytes form large intercellular networks to provide nutritional support for neurons and maintain the dynamic balance of the CNS [51]. Therefore, the dysfunction of astrocytes also resulted in impaired function of glutamate uptake and exacerbated glutamate excitotoxicity [52, 53]. Extracellular glutamate increase has been shown to cause aberrant synaptic signaling that cause neuronal excitotoxicity and death [53]. Our findings demonstrated that c-fos as a rapid and transient marker of activated neurons [54–56] was notably elevated in the spinal cord of rats at the late phase of diabetes. To summarize, significantly decreased astroglia density and activated neurons were exhibited in the spinal cord dorsal horn of rats undergoing DNP at the late phase of diabetes.

The release of TNF-*α*, NF-*κ*B, IL-6, and other proinflammatory factors can be mediated by CXCR4 in the spinal cord dorsal horn, thus increasing neuronal excitability and aggravates central sensitization [57]. In our current work, we found that CXCR4 was mainly present in neurons (Figures 3(a) and 3(b)) and partly in astrocytes, which is in consistence with results of previous researches by us and others [6, 9]. To further investigate the critical role of CXCR4, rats with DNP were intraperitoneally injected with the CXCR4 antagonist AMD3100 at the early and late phases [31]. Intraperitoneal injection of AMD3100 reversed thermal hyperalgesia and mechanical allodynia and downregulated CXCR4 in the spinal cord dorsal horn of rats undergoing DNP at the early phase (Figures 4 and 6(a)) as was reported in other neuropathic pain models [9, 58]. However, AMD3100 did not reverse nociceptive behaviors nor did it regulate the relevant proteins in DNP rats at the late phase. It is speculated that the cascade amplification effect caused by the excessive production of inflammatory factors containing SDF-1 and TNF-*α* may be mediated by CXCR4 stimulation in the spinal cord dorsal horn at the late phase. We evaluated only short-term effects of AMD3100 treatment because of its short half-life (3.6 h) [59]. Another interesting finding is that daily oral administration of NAC, which could reduce nociceptive responses of thermal hyperalgesia and mechanical allodynia in STZ-induced diabetic rats, downregulated CXCR4 expression in DNP rats at the late phase (Figures 4 and 6(b), Figures 3(c)–3(e)). In our previous and current researches, we found that antioxidant NAC treatment reduced oxidative stress (Figure 7) and restored normal autophagic function in STZ-induced diabetic rats [30, 60]. However, daily oral administration of NAC was ineffective in DNP rats in the early phase. A possible explanation is that the treatment duration of one week was too short, and as such, it was not yet effective in the early phase. Similarly, injection of NAC inhibited and reversed chronic inflammatory pain, but it did not reduce nociceptive behavior in the formalin-induced acute pain model in mice [61]. According to current study, CXCR4 exerts an essential role in the peripheral sensitization of STZ-induced diabetic rats with DNP at the early phase. Meanwhile, a persistent increase in CXCR4 and high blood glucose may further exacerbate astrocyte dysfunction at the late phase of diabetes.

### 4.2. Connexin 43 Hemichannel and CXCR4 Release in Astrocytes and Neurons in the Late-Phase Diabetic Neuropathic Pain

CX43 acts as a channel for intercellular connections and allows transmission of ions or small molecule neurotransmitters, which plays a crucial role in neuropathic pain [41, 62–64]. Previous studies showed that CX43 existed in astrocytes [21, 22], while several publications have demonstrated that CX43 existed in neurons [65–67]. Our findings support that the expression of CX43 existed in not only primary cultured astrocytes but also in neurons (Figures 9(c) and 9(d)). In addition, CX43 acts as a channel for intercellular connections and maintains late-phase neuropathic pain [63]. Consistently, our study demonstrated that CX43 was obviously added in the spinal cord dorsal horn of adult rats with DNP in the late phase of diabetes (Figures 8(a)–8(d)). In particular, rats with DNP treated with AMD3100 significantly decreased the expression of CX43 in the spinal dorsal horn. In addition, the finding that the expression of CX43 is regulated by CXCR4 during neuropathic pain is supported by studies conducted in other neuropathic pain models such as nerve ligation [68]. It is speculated that elevation in the expression of proinflammatory CXCR4 occurred in rats with DNP at the late phase of diabetes, and it subsequently induced increases in CX43 expression. CXCL12, a ligand of CXCR4, is likely involved in stimulating the activation and phosphorylation of CX43 via PI3K/Akt pathway, as evidenced in other studies [69, 70]. Furthermore, CXCR4 and CX43 were coexpressed in the spinal cord dorsal horn of STZ-induced DNP rats in the late phase (Figures 9(a) and 9(b)). This suggests that inflammatory or harmful substance-stimulated signals may have been amplified via transmission through CX43 and subsequently facilitated the initiation and the maintenance and exacerbation of DNP. Further study is needed to quantify and locate CX43 in the spinal cord dorsal horn of rodents with DNP and to consolidate its relationship with CXCR4 in the development and progression of DNP.

## 5. Conclusion

**Figure 10:**
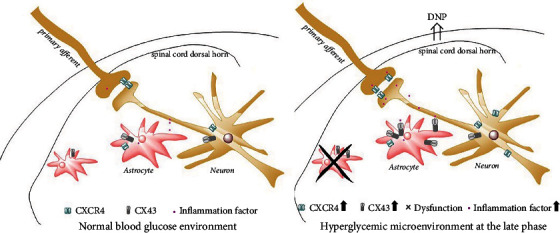
Schematic hypothesis of neuronal-astrocytic CXCR4 and CX43-mediated diabetic neuropathic pain. STZ-induced type 1 diabetes resulted in persistent upregulation of CXCR4 and CX43 in astrocytes and neurons. It is shown that persistent increase of CXCR4 may cause activated neuron excitability and CX43 may mediate intercellular inflammation signal transmission, then dysfunctional astrocytes cannot counter the inflammatory factors at late phase of diabetes, and DNP occurs and exacerbates.

We report unique roles of CXCR4 and CX43 in cell excitability and neurotransmission mediated by dysfunctional astrocytes and activated neurons in the spinal cord of rats with DNP in rats, as summarized in Figure 10. According to results of the present work, the CXCR4/CX43 signaling pathway might play the role of a promising therapeutic target to alleviate DNP. In the late phase, irreversible damage to neurons may be mediated by dysfunctional astrocytes of the spinal cord dorsal horn, which may generate an excitatory neuronal membrane potential and induce an abnormal sense of pain. This dysfunction could be caused by either neuronal damage or inadequate protection of astrocytes, which still requires further exploration.

## Data Availability

The data used to support the findings of this study are available from the corresponding author upon request.
